# Pegzilarginase in Arginase 1 Deficiency: Clinical and Biochemical Effects of Treatment Initiation, Discontinuation and Re-Initiation

**DOI:** 10.3390/children13050610

**Published:** 2026-04-28

**Authors:** Martha Caterina Faraguna, Viola Crescitelli, Roberta Pretese, Maria Valvassori Bolgè, Vera Marchetti, Giusi Sgroi, Stefania Sala, Silvia Gigante, Cristina Bonfanti, Adriana Balduzzi, Serena Gasperini

**Affiliations:** 1 Pediatrics, Fondazione IRCCS San Gerardo dei Tintori, 20900 Monza, Italy; m.faraguna@campus.unimib.it (M.C.F.);; 2 School of Medicine and Surgery, University of Milano-Bicocca, 20900 Monza, Italy

**Keywords:** arginase 1 deficiency, pegzilarginase, recombinant human arginase 1 enzyme, urea cycle disorder, progressive spasticity

## Abstract

**Highlights:**

**What are the main findings?**

- Early dietary restriction may not prevent neurological deterioration in arginase-1-deficiency.
- Real-world data of clinical and biochemical response to pegzilarginase, including biochemical and clinical deterioration after pegzilarginase discontinuation.

**What are the implications of the main findings?**

- Pegzilarginase as a disease-modifying therapy for ARG1-D.

**Abstract:**

Background: Arginase 1 deficiency (ARG1-D) is an ultra-rare urea cycle disorder characterized by hyperargininemia and progressive neurological impairment, including spasticity, loss of motor function, and reduced quality of life. Conventional management based on dietary protein restriction and ammonia scavengers rarely achieves adequate metabolic control or prevents neurological deterioration. Pegzilarginase, a recombinant human arginase 1 enzyme, is the first disease-modifying therapy for ARG1-D. Methods: We report the first Italian real-world experience with pegzilarginase in three pediatric patients with genetically confirmed ARG1-D enrolled in the phase 3 PEACE trial. Clinical, biochemical, functional, nutritional and quality-of-life data were retrospectively collected over a long-term follow-up (2003–2025). Outcomes were evaluated across three phases: treatment initiation (Start), a 13-month treatment interruption due to trial closure (Stop), and therapy re-initiation through an early access program (Restart). Results: Pegzilarginase rapidly normalized plasma arginine levels and was associated with improvements in motor function, spasticity, walking endurance, dietary protein tolerance, bone mineral density, and quality of life. During treatment interruption, all patients experienced biochemical worsening and clinical deterioration, including increased spasticity, reduced mobility, and emotional distress. Re-initiation of pegzilarginase restored metabolic control and led to progressive neurological and functional recovery, including partial reversal of long-standing motor deficits. Conclusions: This real-world experience supports pegzilarginase as a disease-modifying therapy for ARG1-D. Sustained normalization of plasma arginine, rather than subthreshold biochemical control, correlates with functional and neurological improvement and may partially reverse non-lesional metabolic brain injury. Early initiation of pegzilarginase, including in newborn-screened patients, may further modify the natural history of ARG1-D.

## 1. Introduction

Arginase 1 Deficiency (ARG1-D) is an ultra-rare autosomal recessive urea cycle disorder (UCD), caused by biallelic pathogenic variants in the ARG1 gene. The ARG1 gene encodes the hepatic enzyme arginase 1, which catalyzes the final step of the urea cycle by hydrolyzing L-arginine to L-ornithine and urea, an essential reaction for the detoxification of waste nitrogen and the regulation of systemic arginine levels [1,2,3,4,5,6,7]. ARG1-D leads to persistent and marked accumulation of L-arginine in plasma, cerebrospinal fluid and tissues, with intracellular hepatic arginine levels reported up to 50 times above normal. Arginine accumulation leads to increased production of several neurotoxic metabolites and guanidino compounds, initiating a cascade of pathogenic mechanisms, including excessive nitric oxide production, disruption of Na^+^/K^+^-ATPase neuronal function, and impaired GABAergic and glycinergic neurotransmitter signaling, contributing to neuronal injury [3,5].

ARG1-D is characterized by progressive neurological deterioration, and spastic paraparesis, intellectual disability, seizures, and loss of gross motor function are main features [1,6,7]. The estimated prevalence ranges between 1:726,000 and 1:950,000 globally and ~0.61 cases per million in Italy [1,3]. Although ARG1-D is classified among UCDs as a distal defect, its clinical course and pathophysiology are peculiar. Unlike other UCDs, neonatal hyperammonemic encephalopathy is uncommon; symptom onset typically occurs between 1 and 4 years of age, with early signs including tiptoe walking, developmental stagnation or regression, and progressive lower-limb spasticity [1,6,7,8]. Due to its rarity, and phenotypic overlap with cerebral palsy, hereditary spastic paraplegia and hyperornithinemia-hyperammonemia-homocitrullinuria syndrome, ARG1-D is often misdiagnosed or diagnosed late, thereby delaying access to appropriate treatment [1,3]. A timely diagnosis is fundamental to limit, and possibly prevent, brain damage; ARG1-D has been included in Newborn Screening programs in some countries, including Italy, since 2016.

Management of ARG1-D has relied on dietary protein restriction, essential amino acid supplementation, and ammonia scavengers, aiming to reduce plasma arginine levels below 200 µmol/L. However, these strategies are often limited by lack of adherence, poor palatability, growth delay, osteopenia and endogenous arginine production, with most patients failing to achieve therapeutic targets [1,2,6,8,9]. The inadequacy of conventional therapy led to the development of disease-modifying treatments targeting the metabolic pathway of ARG1-D.

Pegzilarginase is a recombinant, pegylated, cobalt-substituted human arginase 1 enzyme designed to restore the deficient enzyme activity in ARG1-D patients. In the Phase 3 Pegzilarginase Effect on Arginase 1 Clinical Endpoints (PEACE) trial, pegzilarginase demonstrated efficacy in normalizing plasma arginine levels and improving motor function. The treatment was well tolerated and enabled patients to exceed guideline-based targets for biochemical control [8,10,11]. In July 2023, pegzilarginase received conditional marketing authorization in the European Union as the first approved pharmacological therapy for ARG1-D, marking a paradigm shift in the clinical management of this disorder [8]. However, data on its real-world use outside of controlled trial settings remain extremely limited, particularly in Europe.

This paper reports the first Italian clinical experience with pegzilarginase, describing the biochemical, neurological and functional outcomes in three pediatric ARG1-D patients, providing preliminary real-world evidence, which can contribute to the improvement of neurological outcome and management of ARG1-D patients. Additionally, we report the exclusive clinical and biochemical effects of a prolonged interruption of treatment on previously treated patients, followed by re-initiation of pegzilarginase.

## 2. Materials and Methods

### 2.1. Setting

Three patients in follow-up at the Fondazione IRCCS San Gerardo dei Tintori, Monza, Italy, were enrolled in the international phase 3 PEACE trial (NCT03921541) since February 2020, first as part of the double-blind phase and then the long-term extension, for a total duration of 35 months. After study completion, treatment was interrupted for 13 months. The patients were subsequently re-started on treatment through an early access program.

### 2.2. Clinical Data

All available data from 2003 to 2025 was retrospectively collected from the clinical charts (both as clinical practice and PEACE study), including

- Biochemical parameters: Plasma arginine, quantified by Ion Exchanged Chromatography, guanidino compounds, was monitored via serial plasma assays during the trial, including argininic acid (ARGA), guanidinoacetic acid (GAA), blood urea nitrogen and ammonia, orotic aciduria. Dedicated blood collection tubes for post-pegzilarginase plasma arginine and guanidino compound assays (e.g., nor-NOHA-specific blood collection tubes) were used to avoid degradation of arginine into the vial.
- Neuromotor and functional assessments: Gross Motor Function Classification System (GMFCS), Gross Motor Function Measure 88 (GMFM-88), Subsections Dimension D (Standing) and E (Walking, Running, Jumping), 2-Minute Walk Test (2MWT), Functional Mobility Scale (FMS).
- Cognitive assessments, including age-appropriate Wechsler Intelligence Scale for Children (WISC).
- Nutritional and bone health parameters, including height, weight, lean body mass index, Dual-Energy X-ray Absorptiometry (DEXA) reports, dietary diary and bromatologic analysis.
- Quality of Life and Functional Autonomy, assessed by Gillette Functional Assessment Questionnaire and Custom QoL questionnaires administered to both families and medical staff. A short 12-items version of the Zarit Burden Interview was used, self-administered by the caregiver (Score range 0–48: 0–10 no to mild burden, 10–20 mild to moderate burden, >20 high burden).

The clinical and biochemical data of the three patients are reported in Table 1.

## 3. Results

### 3.1. Baseline Assessment

#### 3.1.1. Patient 1

Patient 1 was born to consanguineous parents and presented a delayed and complex diagnostic pathway. He achieved autonomous walking at 12 months, although with evident abnormal gait. At 2.5 years, following a febrile episode, he developed tip-toe walking and experienced status epilepticus, initially diagnosed as encephalitis. A disorganized slow-wave activity with theta dominance was identified at electroencephalogram (EE), and treatment with valproic acid was started. Brain magnetic resonance imaging (MRI) revealed T2/FLAIR hyperintensities in the paratrigonal white matter, suggestive of diffuse leukodystrophy. Neurological examination confirmed spastic paraparesis with pronounced hypertonia and lower limb hyposthenia. At 3.5 years, a metabolic workup revealed a plasma arginine level of 470 µmol/L, and subsequent genetic analysis confirmed homozygosity for the missense variant c.749G > A in the ARG1 gene. Over the following years, his clinical course was marked by progression of spasticity, orthopedic complications (Osgood-Schlatter disease), and low bone mineral density (DEXA Z-score −3.9), resulting in several orthopedic surgical interventions and transient loss of ambulation.

Since diagnosis, he was treated with a strict low-protein diet with 0.5 gr/kg of natural protein, amino acids mixture with poor adherence and sodium/glycerol phenylbutyrate, without experiencing hyperammonemic episodes. Despite treatment, arginine was persistently elevated (460–520 µmol/L).

The baseline assessment for PEACE enrollment was performed at age 14. The patient had been wheelchair-dependent for 3 years, was able to walk only short distances with bracing and a walker. A WISC-IV assessment revealed borderline intellectual functioning, with an IQ score of 74.

#### 3.1.2. Patient 2

Patient 2 is the younger sibling of Patient 1. The presence of the same homozygous variant in the ARG1 gene was confirmed soon after birth. Early biochemical testing showed arginine levels of 84 µmol/L at birth, rising to 122 µmol/L by day 10. Despite early diagnosis and dietary intervention since birth (low-protein diet with 0.5 gr/kg of natural protein and amino acids mixture, although with low compliance), her clinical phenotype evolved over time. At age 2, plasma arginine had reached 400 µmol/L and by 2.5 years she displayed spastic diplegia. Seizures emerged by age 3 and were managed with levetiracetam and ethosuximide. Scavengers, sodium phenylbutyrate first, and then glycerol phenylbutyrate, were prescribed and led to good metabolic control in terms of ammonia and glutamine levels.

At PEACE trial baseline, performed at age 8.5 years, she presented with distal paraparesis, rigid hip and tibiotarsal flexors, and an anserine gait requiring orthoses. Bone mineral density was mildly reduced (DEXA Z-score −1.5), and growth was normal. Unlike her brother, she retained partial ambulation, though marked by postural asymmetry and functional limitations, including scissoring gait, tibial stiffness, and lower limb muscle weakness. Borderline intellectual functioning was evident (IQ = 73 assessed by WISC-IV scale).

#### 3.1.3. Patient 3

Patient 3 was born to consanguineous parents. He first showed signs of spastic diplegia and ataxia at age 2; he was diagnosed with ARG1-D at age 2.5 years based on elevated plasma arginine (300–350 µmol/L) and genetic confirmation of a homozygous c.23T > G variant in ARG1. He developed seizures at age 3, which were managed with levetiracetam. He also suffered from pronounced growth retardation, for which growth hormone (GH) therapy was initiated at age 14, resulting in height gain. Despite a strict low-protein diet with 0.5 gr/kg of natural protein and amino acid mixture, arginine levels were persistently high (300–350 µmol/L). In addition, during follow-up, alteration of coagulation and intermittent hypertransaminasemia were noted, with a slight increase in ammonia levels without clinical symptoms.

At baseline assessment for the PEACE trial, performed at age 14, he exhibited bradykinesia, hypomimia, and pronounced spasticity of the lower limbs (scissoring gait, knee valgus, foot pronation). Functional mobility was preserved, albeit limited, with autonomous walking on flat surfaces, achieving only 52 m at baseline in the 2MWT. Cognitive testing, assessed by Wechsler scales, highlighted an IQ score < 40; no adaptive functioning measure was available. The patient also had severe osteopenia (DEXA Z-score −5.8), with a history of pathological fractures.

### 3.2. Pegzilarginase Recombinant Enzyme Therapy

The three patients were enrolled in the PEACE study at age 14, 8.5 and 14, respectively.

The patients initially received pegzilarginase through the PEACE study [8], which was administered intravenously (IV) once weekly, with dosing titrated according to body weight and baseline plasma arginine levels, according to trial protocol [8]. Although the phase I/II study was blinded, it was clinically evident which patient was receiving medication. Despite the COVID-19 pandemic, adherence to treatment was 100%, with no missed doses or protocol violations reported. The patients were then enrolled in the long-term extension (LTE) study.

After 8 weeks of the LTE study, all patients were transitioned to subcutaneous (SC) weekly injections of pegzilarginase. According to the study protocol and manufacturer’s guidelines, the total calculated dose (0.1 mg/kg) was reconstituted and administered in one or two injection sites (abdomen, thighs, or arms), ensuring spacing of at least 3 cm when multiple injections were required. The SC injections were administered at home by a qualified nurse, reducing hospital burden, especially during the COVID-19 pandemic.

After 35 months of treatment and study closure, pegzilarginase administration was suspended for 13 months due to protocol-imposed limitations related to trial closure and regulatory transitions. This phase represented a unique “observation window” during which disease progression in the absence of therapy could be documented.

After 13 months, all three patients were reinitiated on pegzilarginase 0.1 mg/kg subcutaneously under early access authorization. Reintroduction was well tolerated, and the dosing regimen was resumed with the same frequency and formulation as previously established.

#### 3.2.1. Biochemical Response

The biochemical results obtained during the trial have been published [8,10]. In summary, a rapid normalization of plasma arginine was observed, already after only one drug administration.

During the discontinuation period, an increase in plasma arginine levels was observed in all three patients (Table 1). The patients were started, similarly to before trial enrollment, on a strict diet and the dosage of scavengers was increased.

After treatment re-initiation, a rapid biochemical response to treatment was observed once again, consistently achieving concentrations well below the therapeutic target of <200 µmol/L (Table 1).

Plasma guanidinoacetic acid (GAA), a neurotoxic biomarker, was measured only in Patient 3. It was elevated at baseline (2.07 µmol/L), later increased to 6.73 µmol/L during the treatment interruption, then normalized upon pegzilarginase re-initiation.

#### 3.2.2. Neurological Response

The positive benefits of treatment with pegzilarginase during the clinical trial have been previously published [8,10]; in summary, improvement of gross motor function, walking distance, and functional mobility were observed, as well as a reduction in spasticity.

After treatment discontinuation, clinical deterioration was documented in all three patients, in particular an increase in spasticity and motor function decline (Table 1). Patient 1 underwent bilateral orthopedic surgery due to worsening contractures (calcaneal valgus osteotomy and talonavicular arthrodesis) and completely lost residual ambulatory ability. Patient 2 exhibited recurrence of scissoring gait, postural asymmetry, and tibiotarsal stiffness. Patient 3 showed decreased endurance, and relapse of hypomimia and bradykinesia.

**Figure 1:**
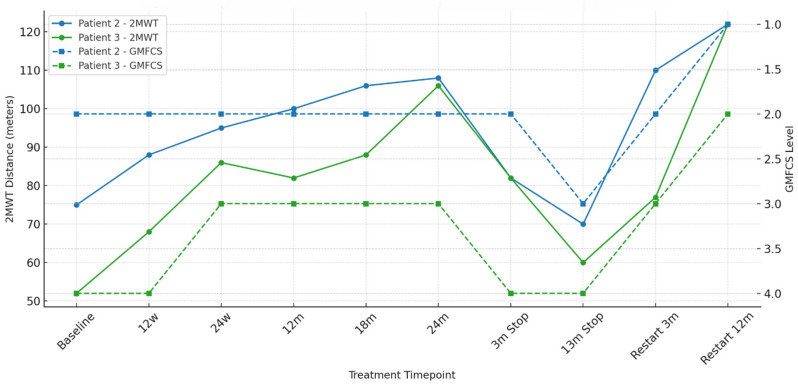
Motor performance, assessed by Two-minute Walk Test (2MWT), and motor disability level, assessed by Gross Motor Function Classification System (GMFCS) in Patients 2 and 3. The impact of pegzilarginase interruption and reinitiation is clearly visible, with a transient clinical deterioration followed by a marked functional recovery. The GMFCS axis is inverted to accurately reflect that lower scores correspond to better motor function. Abbreviations: w = weeks; m = months.

After treatment re-initiation, Patient 1, initially wheelchair-dependent, regained partial lower-limb extension and improved upper-limb coordination. Despite persistent orthopedic limitations post-surgery, improvements were measurable in fine motor control and functional reach. During the last evaluation at age 19 years, 18 months after restarting therapy, he was able to walk for few steps alone with a leg brace, after many years of using a wheelchair. Patient 2 demonstrated the most striking motor improvement, progressing from GMFCS level II to I, achieving full ambulation, stair climbing without support, and even running on uneven surfaces. The 2MWT improved by 62% from baseline (Table 1, Figure 1). Spasticity diminished significantly, with scissoring gait nearly abolished. Patient 3 showed quantitative and qualitative gains, including a >50% increase in 2MWT distance (Table 1, Figure 1). During clinical examination, improvements in hypomimia, bradykinesia, and proximal lower-limb strength were documented, contributing to a smoother gait and better endurance, more understandable speech and more social interaction with peers. After restarting therapy, he continued to improve over time. At last evaluation, after 18 months from restarting enzyme, he could climb the stairs without support for the first time in his life.

#### 3.2.3. Skeletal and Mineralization Outcomes

Bone mineral density, assessed by DEXA, improved under treatment. After three years of treatment, the total body bone density z-score increased from −3.9 to −2.2, and from −2.5 to −2 in Patients 1 and 2, respectively. Patient 3 had a history of bone fracture before starting treatment; his bone mineral density increased from −5.8 to −4.2. In addition, an increase in lean mass was observed. All patients concurrently received vitamin D supplementation.

#### 3.2.4. Dietary Treatment

During the treatment periods (PEACE study and re-initiation), the dietary restrictions were loosened. Gradually, the patients began eating a broader variety of foods, which translated into greater satisfaction, reduced mealtime conflict, and, especially in adolescence, a sense of normalization and inclusion in family life. After 6 months of treatment, all three patients were able to safely gradually increase their natural protein intake by 20–40%, with normalized plasma arginine levels. From a clinical nutrition perspective, improved nitrogen balance, stabilization of energy intake, and enhanced intake of micronutrients (e.g., calcium, phosphorus, zinc) contributed to improved body composition and bone mineral accrual, particularly evident in DEXA improvements in Patient 1 and Patient 3. As of the latest follow-up of treatment re-initiation, protein intake was maintained at approximately 1.2 g/kg/day, with a progressive tapering of synthetic essential aminoacids (EAA) supplementation. Scavenger use was optimized according to glutamine level but not discontinued. Appetite, growth, and muscle mass accrual improved, particularly in Patient 3, whose height trajectory reached the 3rd percentile following combined pegzilarginase and GH therapy.

#### 3.2.5. Quality of Life Measures

Treatment with pegzilarginase lead to a multidimensional improvement in quality of life (QoL), as perceived by patients, caregivers, and the multidisciplinary medical team.

#### 3.2.6. Caregiver-Reported Outcomes

**Figure 2:**
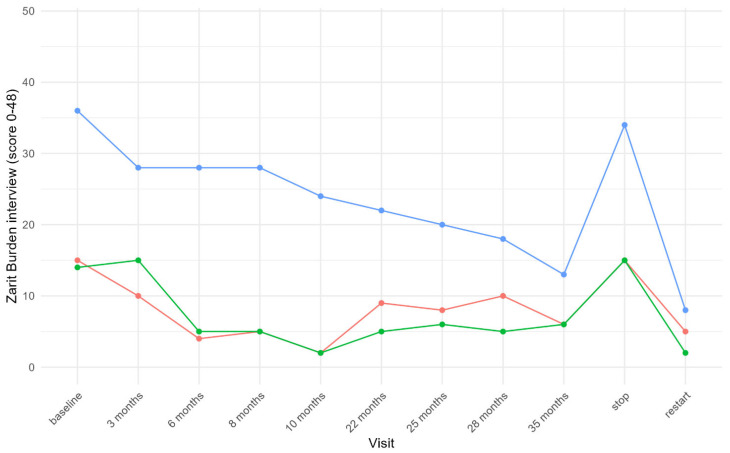
Results of the Zarit Scale of Caregiver Burden assessed during the three phases. During the trial a short 12-items version was used, self-administered by the caregiver. Total score range 0–48 (0–10: no to mild burden, 10–20: mild to moderate burden, >20: high burden). Each patient is represented by a different color.

During the treatment periods (PEACE study and re-initiation), caregivers reported a reduction in physical dependency for basic and instrumental activities of daily living (ADLs), including walking unaided, dressing, stair climbing, and toileting. Moreover, improvements in mood, emotional regulation, and self-confidence were frequently cited. One parent described their child as “finally able to feel like other adolescents”, a sentiment echoed in all families. In two patients, caregivers observed increased concentration, improved school performance, and greater participation in classroom activities, reflected also in patient-reported satisfaction. The Zarit Burden Interview scores after starting pegzilarginase decreased in all families, indicating lower perceived stress levels, especially after transitioning to subcutaneous home therapy (Figure 2).

#### 3.2.7. Patient-Reported Outcomes (PROs)

Patient 2 and 3, with preserved insight and communicative capacity, confirmed the positive impact of therapy. Patient 2 described being able to engage in activities previously impossible, such as running on uneven surfaces, climbing stairs independently, and participating in peer social interactions. She emphasized the emotional significance of “not needing to be helped all the time”. Patient 3 expressed greater affectivity, cooperation, and motivation during physiotherapy sessions, as reported by clinicians and therapists.

#### 3.2.8. Clinician Observations and Multidisciplinary Consensus

Medical staff completed a structured questionnaire every 3–6 months, documenting perceived changes in the following domains: physical performance, which was rated “greatly improved” in 2 of 3 patients; neurocognitive responsiveness; emotional reactivity and social engagement; ease of therapeutic management and communication; and general impression of well-being. The care team unanimously reported that pegzilarginase therapy yielded improvements that were “visually perceptible”, particularly in motor fluency, interaction, and cooperative behavior during rehabilitation sessions.

## 4. Discussion

We hereby described three cases of ARG1-D patients who received enzyme therapy with pegzilarginase. Its administration led to a normalization of plasma arginine levels, a loosening of dietary restrictions and medication use, an improvement of motor and neurological outcome, and was also associated with a better quality of life both for caregivers and patients.

Importantly, the present study provides insight into the clinical and biochemical effects of treatment discontinuation. The interruption of pegzilarginase for 13 months led to a loss of previously acquired clinical gains and a stressful period for the families. While recovery from the orthopedic surgery may have contributed to the clinical decline observed for Patient 1 during treatment discontinuation, no other factors were found for the other two patients. After a year of drug re-administration, the motor and neurological benefits were persistently regained.

The medication was well tolerated and no serious adverse events were reported. A single patient experienced a mild injection site reaction, which resolved without intervention. Home therapy proved to be safe and feasible, even in patients with significant neurodevelopmental comorbidities, after appropriate caregiver training. The subcutaneous home administration was greatly appreciated by the patients and their families, as observed for other enzyme replacement therapies [12,13].

We hereby reported the results of treatment in long-time symptomatic patients: neurological improvement in patients with apparently consolidated disabilities was surprising and unexpected. It will be interesting to see the effects of pegzilarginase on the course of the disease if treatment is started in pre-symptomatic patients. To date, in Italy, it is possible to detect ARG-1-D through newborn screening; timely diagnosis has determined prompt intervention with dietary restriction and the administration of scavengers. Unfortunately, as seen for Patient 2, such interventions are not sufficient in preventing the onset of spasticity. If administered before neurotoxic arginine accumulation begins, pegzilarginase could potentially prevent or greatly reduce neurodevelopmental impairment, altering the natural history of ARG1-D [5].

Clinical benefits of treatment were not only seen in neurological terms. Indeed, administration of pegzilarginase, and the subsequent loosening of dietary restrictions, led to improved bone health and body composition. Dietary restrictions prescribed for many inborn errors of metabolism are often associated with osteopenia and osteoporosis [14]; bone fractures may contribute to limited mobility, as seen in the patients reported. Thus, treatment with pegzilarginase may have the potential to address a key feature of long-term consequences of dietary management in ARG1-D.

The Phase 3 PEACE trial [8] previously demonstrated that true clinical efficacy was associated with the achievement of plasma arginine concentrations within the physiological range (40–115 µmol/L), which was reached in over 90% of patients treated with pegzilarginase. While historical management guidelines defined plasma arginine levels below 200 µmol/L as the therapeutic target to reduce the risk of neurotoxicity, we could hypothesize that the therapeutic goal could potentially be implemented to the sustained normalization of plasma arginine, even under a progressively liberalized diet.

This study reflects the experience of a single center treating three patients. The findings are limited by the retrospective nature of data collection from clinical charts. Moreover, cognitive testing was not performed either following treatment initiation or during its discontinuation. Despite the small size, the clinical observations are in line with a recently published real-world French cohort [15]. International collaborative studies are necessary to evaluate the effects of treatment in a large cohort of ARG1-D patients.

## 5. Conclusions

In conclusion, we have learnt a lot from our patients and from the three-phase experience (start/stop/restart therapy): early treatment relying solely on dietary restriction does not prevent neurological deterioration; it is not too late to initiate therapy in complex chronic patients; and neurological impairment with severe spasticity may be ameliorated by pegzilarginase, and the clinical benefit may continue to improve over time in parallel with the normalization of arginine levels and GAA profiles.

Similarly to what has been observed in phenylketonuria, the correction of biochemical parameters, in this case plasma arginine, appears to drive the improvement of functional deficits through the restoration of neuromodulator homeostasis, with a plausible positive impact on neurotransmitter balance, synaptic function, autophagic activity, neuroinflammation and oxidative cellular status. This evidence supports the concept that non-lesional neurological damage due to chronic metabolic imbalance can be, at least in part, reversible through metabolic correction.

## Figures and Tables

**Table 1:** Clinical, biochemical and functional outcomes in three Italian pediatric patients with ARG1-D before and after pegzilarginase therapy. Data at baseline (Pre) and at last evaluation (Post) is reported. Abbreviations: ARG1-D—Arginase 1 Deficiency; GAA—Guanidinoacetic Acid; GMFCS—Gross Motor Function Classification System; 2MWT—2-Minute Walk Test; DEXA—Dual-Energy X-ray Absorptiometry; EAA—Essential Amino Acids; VPA—Valproic Acid; LEV—Levetiracetam; ETS—Ethosuximide; FMS—Functional Mobility Scale; QoL—Quality of Life, n/a—not available.

Parameter	Patient 1 (Pre-Treatment)	Patient 1 (Post-Treatment)	Patient 2 (Pre-Treatment)	Patient 2 (Post-Treatment)	Patient 3 (Pre-Treatment)	Patient 3 (Post-Treatment)
**Plasma arginine (µmol/L)**	460–520	105	300–400	100–150	300–350	<120
**GAA (µmol/L)**	n/a	n/a	n/a	n/a	6.73 (during stop phase)	Normalized
**Ammonia**	Normal	Normal	Normal	Normal	Slightly increased (no symptoms)	Normal
**Dietary protein intake (g/kg/day)**	0.5 EAA supplementation	1.0–1.2	0.5	1.0–1.2	0.5	1.0–1.2
**Scavenger therapy**	Sodium Benzoate/glycerol phenylbutyrate	Reduced dose	Sodium Benzoate/glycerol phenylbutyrate	Reduced dose	Glycerol phenylbutyrate	Reduced dose
**Seizure control**	Controlled on VPA	Stable, no relapse	Controlled on LEV/ETS	Stable, no relapse	Controlled on LEV	Stable
**Spasticity/Motor phenotype**	Severe paraparesis, wheelchair dependent	Partial lower-limb extension, walks a few steps, improved trunk control	Spastic diplegia, scissoring gait	Fully ambulatory, smooth gait	Bradykinesia, hypomimia, spastic diplegia	Reduced bradykinesia, smoother gait, climbs the stairs with support
**GMFCS level**	IV	III	II	I	IV	II–III
**Functional mobility (FMS)**	Wheelchair, assisted walking	Able to walk few steps with brace	Orthoses, limited ambulation	Independent walking, stair climbing	Limited ambulation: scoring 1 at 500 m	Increased endurance: scoring 5 at 500 m
**Postural/facial control**	Hypotonic, trunk instability	Improved trunk and hand control	Asymmetric posture	Symmetric, improved balance	Hypomimia	Facial reactivity restored
**2MWT (meters)**	Not testable	10	75	122 (+62%)	52	122 (+134%)
**Total body DEXA Z-score**	−3.9	−2.2	−1.5	−1.1	−5.8	−4.2
**Fractures**	None	None	None	None	History of fractures	No new fractures
**Quality of Life (QoL)**	Low; high caregiver burden	Improved motivation, socialization	Anxiety, motor frustration	Increased autonomy, mood stability	Dependent, limited affectivity	Improved engagement, cooperation
**School/Social function**	Poor participation	Moderate, increased engagement	Limited, physical barriers	Full participation	Poor	More cooperative, attentive
**Adverse events**	-	Mild injection site erythema on one occasion	-	None	-	None

## Data Availability

All original data is present in the manuscript. Further enquiries can be addressed to the corresponding author.
